# Stiff person syndrome in South Asia

**DOI:** 10.1186/s13104-016-2276-z

**Published:** 2016-10-18

**Authors:** Thashi Chang, Bethan Lang, Angela Vincent

**Affiliations:** 1 Department of Clinical Medicine, Faculty of Medicine, University of Colombo, 25, Kynsey Road, Colombo, 00800 Sri Lanka; 2 Nuffield Department of Clinical Neurosciences, John Radcliffe Hospital, Level 5/6 West Wing, Oxford, OX3 9DU UK

**Keywords:** Stiff person syndrome, GAD antibodies, Autoimmune, CNS, Sri Lanka, Case report

## Abstract

**Background:**

Stiff person syndrome is a highly disabling, progressive autoimmune disorder of the central nervous system characterized by muscle rigidity and spasms. Stiff person syndrome is rare, but is believed to be under diagnosed with only 14 cases been reported among a 1.7 billion population in South Asia. We report the first authenticated case from Sri Lanka.

**Case presentation:**

A 55-year-old Sri Lankan female presented with difficulty in walking and recurrent falls due to progressive muscular rigidity in her lower limbs and trunk with superimposed muscle spasms that occurred in response to unexpected noise, startle or emotional upset. She had anxiety and specific phobias to open spaces, walking unaided and being among crowds of people. She had insulin-dependent diabetes mellitus and was on thyroxine replacement. On examination, she had hyperlordosis combined with board-like rigidity of her anterior abdomen and rigidity of her lower limbs bilaterally. Upper limbs were normal. Magnetic resonance imaging of her neuraxis was normal. Electromyography showed continuous motor unit activity at rest. Glutamic acid decarboxylase antibodies were detected in her serum at a titre of 15,500 IU/ml (normal <5). She showed a remarkable and sustained improvement to treatment with intravenous immunoglobulins, immunosuppressive and muscle relaxant medications, regaining independent ambulation.

**Conclusions:**

Diagnosis of stiff person syndrome remains clinical, supported by electromyography and serology for glutamic acid decarboxylase antibodies, facilitated by a high index of clinical suspicion. An autoimmune basis lends stiff person syndrome amenable to treatment highlighting the importance of diagnosis. This case adds to map the worldwide distribution of stiff person syndrome.

## Background

Stiff person syndrome (SPS), first described by Moersch and Woltman in 1956, is a rare, highly disabling, progressive autoimmune disorder of the central nervous system (CNS) characterized by muscle rigidity and spasms [1]. Since its original description, several variants of the syndrome including stiff limb syndrome, jerking SPS, paraneoplastic SPS and progressive encephalomyelitis with rigidity and myoclonus have been described [2]. High titres of autoantibodies to glutamic acid decarboxylase (GAD), the rate-limiting enzyme in the synthesis of the inhibitory neurotransmitter gamma-amino butyric acid (GABA), have been reported in approximately 60–80 % of patients with classic SPS [3]. However, SPS remains a clinical diagnosis facilitated by a high index of suspicion. SPS has an estimated prevalence of 1/million population [4], but is believed to be underdiagnosed [5]. Thus far, only 14 cases have been reported from South Asia, which has a collective population of over 1.7 billion. We report the first authenticated case of classic SPS from Sri Lanka.

## Case presentation

A 55-year-old Sri Lankan female presented with progressive difficulty in walking superimposed with muscle spasms since 2002. She had first noted intermittent stiffness in her right lower limb which lasted about 5–10 min at a time but gradually, over months, increased in frequency and duration to be persistent. She then noted the stiffness spreading to involve her left lower limb making ambulation increasingly difficult. From about 2009, the muscles of her trunk and lower limbs would go into severe painful spasms in response to unexpected noise, startle or emotional upset. These spasms initially occurred about 1–2/month and lasted about 30–60 min but over time increased in both frequency and duration. These episodes were associated with drenching sweats and fear. She has had several falls and injuries due to sudden muscle spasms. She also reported generalised anxiety since the onset of the illness in 2002 and specific phobias to open spaces, walking unaided and being among a crowd of people. She had felt depressed at times which she attributed to the disabling nature of the illness which had curtailed her independence. In 2007, she was diagnosed with diabetes mellitus, which required insulin to achieve normal glycaemic control. In 2014, she was found to be biochemically hypothyroid and commenced on thyroxine replacement. She has had 3 repeated magnetic resonance imaging (MRI) of the brain and spinal cord since 2002, all of which were reported as normal. After extensive investigation she had been given a presumptive diagnosis of ‘non-compressive myelopathy’ of unknown aetiology and treated with diazepam and baclofen. However, her condition continued to deteriorate with time.

**Fig. 1:**
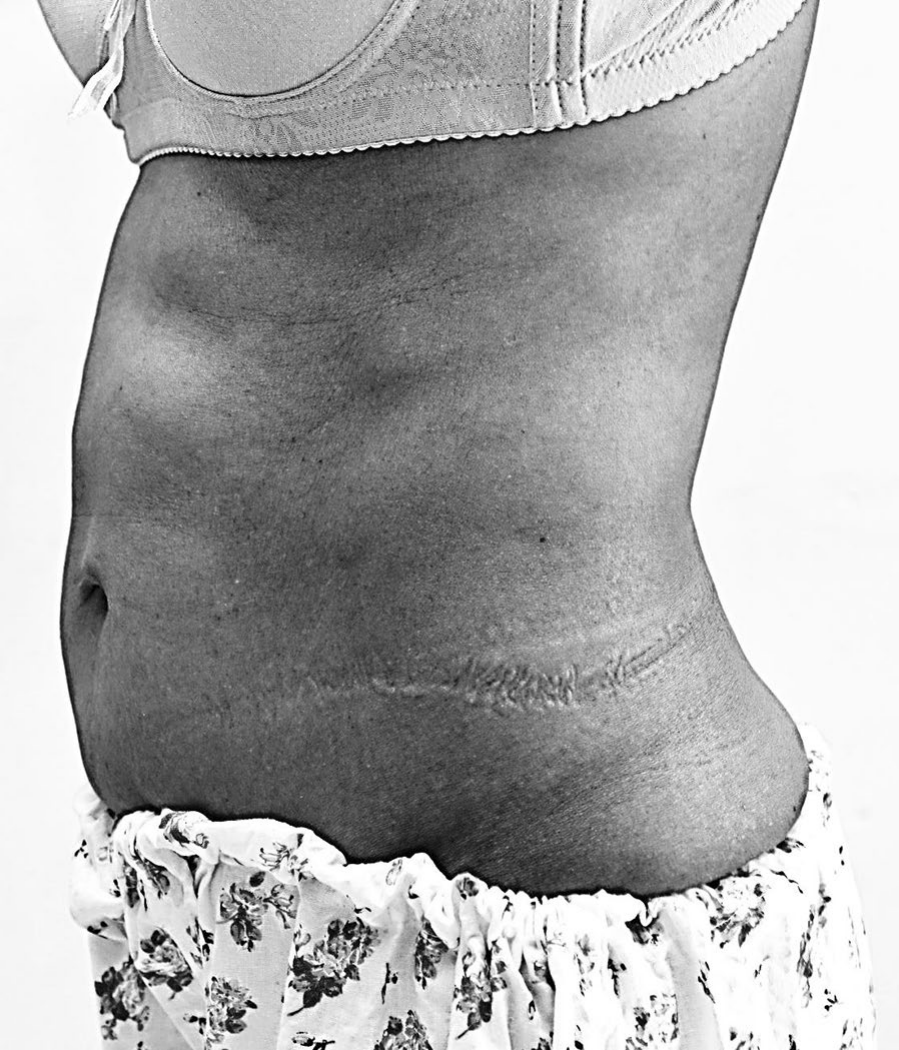
Hyperlordosis of lumbar spine associated with rigid anterior abdominal wall muscles demonstrated by prominently defined rectus abdominis

On examination in 2015 she had hyperlordosis of the lumbar spine (Fig. 1) due to rigid contraction of her thoracolumbar paraspinal muscles associated with stony-hard, board-like rigidity of her anterior abdominal muscles, markedly increased tone in both lower limbs, and more on the left with the left ankle being in a plantarflexed posture. Clonus was absent. Muscle power was 5/5 on the right and 4+/5 on the left. Knee jerks were brisk, ankle jerks were normal and the plantar responses were flexor. The sensory examination was normal. Her distribution of stiffness index was 3/6 (number of stiff areas, range 0–6) and heightened sensitivity index was 7/7 (number of stimuli that induce muscle spasms, range 0–7) while the modified Rankin score (mRS, range 0–6) was 4. Examination of her upper limbs and cranial nerves were normal. General and other system examinations were normal. Her blood pressure was 130/80 mmHg.

Routine haematological and biochemical investigations including blood counts, inflammatory markers, liver function and renal function tests were normal. Blood glucose was maintained within normal limits with subcutaneous insulin. Her thyroid stimulating hormone level had been over 100 miU/ml before commencement of thyroxine. Anti-thyroglobulin antibodies were 102.16 IU/ml (normal 0.00–4.11). Anti-thyroid peroxidase antibodies were 1076 IU/ml (normal 0.5–5.6). Antinuclear antibodies and rheumatoid factor were normal. Anti-GAD antibodies were 15,500 IU/ml (normal <5). Electromyography (EMG) of abdominal and paraspinal muscles showed continuous motor unit activity (CMUA) at rest. Nerve conduction studies were normal.

She was treated with intravenous immunoglobulin (IVIg) 0.4 g/kg/d for 5 days and commenced on levetiracetam 250 mg BD later increased to 500 mg BD, mycophenolate mofetil 250 mg BD later increased to 500 mg BD and increasing doses of diazepam, baclofen and tizanidine. Humulin insulin and thyroxine were titrated to achieve normoglycemia and normal TSH levels. Medical treatment was combined with physiotherapy. Her distribution of stiffness index reduced to 1/6 after 1 month and the heightened sensitivity index reduced to 1/7 after 3 months while the mRS improved to 3 after 3 months and has remained that at 1 year follow up. Additionally, her susceptibility to excessive startle and anxiety resolved with treatment, but she continued to have agoraphobia and phobia to open spaces, albeit to a lesser degree. She had a second course of IVIg, 6 months after the initial treatment and has remained clinically stable on immunosuppressive and symptomatic medication.

## Discussion

SPS is most common in the fourth and fifth decades of life affecting more women than men [5, 6]. Our patient fulfilled the clinical criteria of SPS which includes (1) muscular rigidity in limbs and axial muscles; (2) continuous co-contraction of agonist and antagonist muscles; (3) episodic spasms precipitated by unexpected noises, tactile stimuli or emotional upset; (4) absence of any other neurological disease that could explain stiffness and rigidity; and (5) presence of anti-GAD antibodies (GAD-Abs) in serum [7]. She had increased paraspinal muscle tone causing hyperlordosis, which is a hallmark clinical sign of SPS (Fig. 1). The diagnosis in our patient was clinically supported by the characteristic, but not unique, EMG finding of CMUA and the detection of serum GAD-Abs in high titre. GAD-Abs are most commonly detected in type 1 diabetes mellitus (T1DM), which occurs in about 40 % of patients with SPS as seen in our patient, but the titre in T1DM is markedly lower than that of SPS [8]. Although high titres of GAD-Ab in the range seen in SPS (>1000 IU/ml) have been reported in some cases of cerebellar ataxia and epilepsy [8, 9], the prevalence of GAD-Abs in those diseases is rare. Other organ specific autoimmune diseases and autoantibodies, such as the thyroid disease in our patient, are found in around 40 % of patients with SPS [6].

A very typical but poorly appreciated feature is the marked anxiety, paroxysmal fear, excessive startle and specific phobias such as of open spaces that are frequently found in these patients. The rate of specific phobia in SPS is at least five times greater than in the general population [10]. These symptoms coupled with emotionally triggered spasms often contribute to the initial misdiagnosis as neurosis and a delay in the diagnosis of SPS. There was a delay in diagnosis of 12 years in our patient, possibly due to the slow evolution of the disease coupled with a low index of suspicion. The average time from symptom onset to diagnosis has been reported to be 6.2 years (range 1–18 years) [6].

SPS remains a clinical diagnosis supported by typical EMG findings and positive serology for GADAbs. Other causes of rigidity and spasms need exclusion. These include chronic tetanus, neuromyotonia, dystonia, hyperekplexia with late presentation, spinal multiple sclerosis or paraneoplastic myelitis, intrinsic spinal cord tumours or syrinx and psychologically determined disorders. Our patient presented with typical features of SPS supported with EMG features and high-titre GAD-Abs. There was no clinical or neurophysiological evidence of hyperekplexia, dystonia, myotonia or upper motor neuron syndromes. However, the severe rigidity of her limbs had been misdiagnosed as spasticity even though she had not had upper motor neuron signs such as extensor plantar responses, clonus or a sensory level, thus contributing to a delay in diagnosis and appropriate treatment.

The clinical severity of SPS can be graded according to the distribution of stiffness index, which records the number of stiff areas ranging from 0 (none) to 6 and the heightened sensitivity index, which records the number of stimuli that induces spasms ranging from 0 (none) to 7 [6]. Our patient showed remarkable and sustained improvement of both these indices and mRS few months after initiating immunomodulatory and immunosuppressive therapies. IVIg is the only immunomodulatory treatment that has been shown to confer significant improvement in a randomised, double-blinded, placebo-controlled crossover trial [11].

It has been suggested that GAD-Abs are pathogenic and cause loss of GABAergic inhibition in the CNS [12] that is responsible for the muscle spasms and rigidity. However, given that GAD is an intracellular protein that is not thought to be accessible to circulating antibodies, it may be that GAD-Abs are part of a wider autoimmune process rather than the specific cause of the disease [13, 14].

## Conclusions

Our case report highlights the need for a high index of clinical suspicion in order to diagnose a rare, but progressively disabling autoimmune central nervous system disorder for which effective treatment is available. It further adds to map the worldwide distribution of SPS in different populations.

## Abbreviations

Abs: antibodies
CMUA: continuous motor unit activity
CNS: central nervous system
EMG: electromyogram
GABA: gamma-amino butyric acid
GAD: glutamic acid decarboxylase
IVIg: intravenous immunoglobulin
MRI: magnetic resonance imaging
mRS: modified Rankin scale
SPS: stiff person syndrome
TSH: thyroid stimulating hormone
